# Dichloridobis(4-methyl-3,5-diphenyl-1*H*-pyrazole-κ*N* ^2^)copper(II)

**DOI:** 10.1107/S1600536811050690

**Published:** 2011-11-30

**Authors:** Moayad Hossaini Sadr, Behzad Soltani

**Affiliations:** a Research Institute for Fundamental Sciences (RIFS), University of Tabriz, 51664, Tabriz, Iran; b Department of Chemistry, Azarbaijan University of Tarbiat Moallem, Tabriz, Iran

## Abstract

The asymmetric unit of the title compound, [CuCl_2_(C_16_H_14_N_2_)_2_], comprises half of the complex. The Cu^II^ atom lies on a crystallographic twofold rotation axis and shows a significantly distorted tetra­hedral coordination geometry. The dihedral angle between the phenyl rings is 74.3 (2)°. The crystal structure is stabilized by inter­molecular π–π inter­actions [centroid–centroid distances = 3.635 (2)–3.803 (3) Å].

## Related literature

For standard bond lengths, see: Allen *et al.* (1987 ▶). For background to pyrazole chemistry, see: Mukherjee (2000 ▶); Mukherjee & Sarka (2003 ▶); Hossaini Sadr *et al.* (2004 ▶, 2005 ▶).
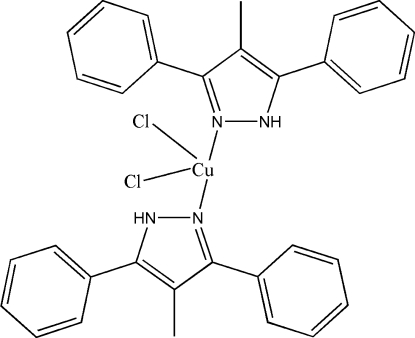


## Experimental

### 

#### Crystal data

[CuCl_2_(C_16_H_14_N_2_)_2_]
*M* *_r_* = 603.03
Monoclinic, 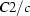

*a* = 19.105 (4) Å
*b* = 8.5062 (17) Å
*c* = 17.399 (4) Å
β = 99.39 (3)°
*V* = 2789.6 (11) Å^3^
*Z* = 4
Mo *K*α radiation
μ = 1.00 mm^−1^
*T* = 120 K
0.23 × 0.09 × 0.03 mm

#### Data collection

Stoe IPDS II Image Plate diffractometer
Absorption correction: numerical (*X-RED*; Stoe & Cie, 2005 ▶) *T*_min_ = 0.895, *T*_max_ = 0.970
9857 measured reflections
3752 independent reflections
3026 reflections with *I* > 2σ(*I*)
*R*_int_ = 0.119

#### Refinement

*R*[*F* ^2^ > 2σ(*F* ^2^)] = 0.074
*wR*(*F* ^2^) = 0.215
*S* = 1.11
3752 reflections
181 parameters
H atoms treated by a mixture of independent and constrained refinement
Δρ_max_ = 0.87 e Å^−3^
Δρ_min_ = −0.76 e Å^−3^

### 

Data collection: *X-AREA* (Stoe & Cie, 2005 ▶); cell refinement: *X-AREA*; data reduction: *X-AREA*; program(s) used to solve structure: *SHELXS97* (Sheldrick, 2008 ▶); program(s) used to refine structure: *SHELXL97* (Sheldrick, 2008 ▶); molecular graphics: *SHELXTL* (Sheldrick, 2008 ▶); software used to prepare material for publication: *PLATON* (Spek, 2009 ▶) and *SHELXTL*.

## Supplementary Material

Crystal structure: contains datablock(s) global, I. DOI: 10.1107/S1600536811050690/rz2665sup1.cif

Structure factors: contains datablock(s) I. DOI: 10.1107/S1600536811050690/rz2665Isup2.hkl

Additional supplementary materials:  crystallographic information; 3D view; checkCIF report

## Figures and Tables

**Fig. 1.**
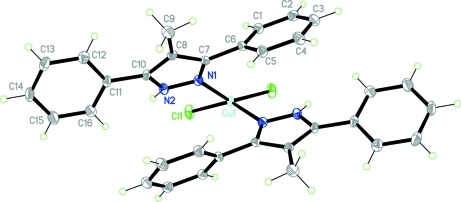
The molecular structure of the title compound, showing 50% probability displacement ellipsoids. Unlabelled atoms are related to the labelled atoms by the symmetry operation 1-x, y, 1/2-z.

## supplementary crystallographic information

### Comment

There are a lot of publications on coordination chemistry of pyrazole-based chelating ligands which present versatile coordination geometry and nuclearity (Mukherjee, 2000; Mukherjee & Sarka, 2003). The suitable structure and high stability of pyrazoles, in addition to the ability of their deprotonated form to act as powerful nucleophiles in substitution reactions, have made them good candidates for incorporation in the design of new ligands. The easy control of the electronic and steric properties of the pyrazolyl-derived ligands by introducing different substituents in the pyrazolyl rings is another advantage and expands the domain of pyrazole-type ligands. As part of a general study of pyrazole ligands (Hossaini Sadr et al., 2005; Hossaini Sadr et al., 2004), we have determined the crystal structure of the title compound.

The asymmetric unit of the title compound, Fig. 1, comprises half of the complex. The copper(II) atom lies on a crystallographic two-fold rotation axis and shows a significantly distorted tetrahedral coordination geometry. The dihedral angle between the phenyl rings is 74.3 (2)°. The crystal structure is stabilized by intermolecular π-π interactions [Cg1···Cg2^i^ = 3.635 (2)Å, Cg3···Cg3^ii^ = 3.803 (3)Å; Cg1, Cg2 and Cg3 are centroids of the N1/N2/C10/C8/C7, C1–C6, and C11–C16 rings, respectively; symmetry codes: (i) 1-x, y, 1/2-z, (ii) 1/2-x, 1/2-y, -z].

### Experimental

The title compound was synthesized by adding dry CuCl_2_ (1 mmol, 134 mg) to a solution of 4-methyl-3,5-diphenyl-1*H*-pyrazole (2 mmol, 468 mg) in THF (30 ml). The mixture was stirred for 12 hour. The resultant yellow solution was filtered and the solid phase washed by ether and dried in *vacuo*. Orange single crystals of the title compound suitable for *X*-ray structure determination were recrystallized from acethonitrile by slow evaporation of the solvent at room temperature over several days.

### Refinement

The N-bound atoms was located in a difference Fourier map and refined freely. All other H atoms were positioned geometrically and refined using a riding model, with C—H = 0.93–0.96 Å and with *U*_iso_(H) = 1.2*U*_eq_(C) or 1.5*U*_eq_(C) for methyl H atoms.

### Crystal data

**Table:**

[CuCl_2_(C_16_H_14_N_2_)_2_]	*F*(000) = 1244
*M**_r_* = 603.03	*D*_x_ = 1.436 Mg m^−^^3^
Monoclinic, *C*2/*c*	Mo *K*α radiation, λ = 0.71073 Å
Hall symbol: -C 2yc	Cell parameters from 2500 reflections
*a* = 19.105 (4) Å	θ = 2.5–28.4°
*b* = 8.5062 (17) Å	µ = 1.00 mm^−^^1^
*c* = 17.399 (4) Å	*T* = 120 K
β = 99.39 (3)°	Needle, orange
*V* = 2789.6 (11) Å^3^	0.23 × 0.09 × 0.03 mm
*Z* = 4	

### Data collection

**Table:**

Stoe IPDS II Image Plate diffractometer	3752 independent reflections
Radiation source: fine-focus sealed tube	3026 reflections with *I* > 2σ(*I*)
graphite	*R*_int_ = 0.119
Detector resolution: 0.15 mm pixels mm^-1^	θ_max_ = 29.3°, θ_min_ = 2.2°
rotation method scans	*h* = −26→26
Absorption correction: numerical (*X-RED*; Stoe & Cie, 2005)	*k* = −11→10
*T*_min_ = 0.895, *T*_max_ = 0.970	*l* = −23→21
9857 measured reflections	

### Refinement

**Table:**

Refinement on *F*^2^	Primary atom site location: structure-invariant direct methods
Least-squares matrix: full	Secondary atom site location: difference Fourier map
*R*[*F*^2^ > 2σ(*F*^2^)] = 0.074	Hydrogen site location: inferred from neighbouring sites
*wR*(*F*^2^) = 0.215	H atoms treated by a mixture of independent and constrained refinement
*S* = 1.11	*w* = 1/[σ^2^(*F*_o_^2^) + (0.0975*P*)^2^ + 21.982*P*] where *P* = (*F*_o_^2^ + 2*F*_c_^2^)/3
3752 reflections	(Δ/σ)_max_ = 0.004
181 parameters	Δρ_max_ = 0.87 e Å^−^^3^
0 restraints	Δρ_min_ = −0.76 e Å^−^^3^

### Special details

**Table:**

Geometry. All esds (except the esd in the dihedral angle between two l.s. planes) are estimated using the full covariance matrix. The cell esds are taken into account individually in the estimation of esds in distances, angles and torsion angles; correlations between esds in cell parameters are only used when they are defined by crystal symmetry. An approximate (isotropic) treatment of cell esds is used for estimating esds involving l.s. planes.
Refinement. Refinement of F^2^ against ALL reflections. The weighted R-factor wR and goodness of fit S are based on F^2^, conventional R-factors R are based on F, with F set to zero for negative F^2^. The threshold expression of F^2^ > 2sigma(F^2^) is used only for calculating R-factors(gt) etc. and is not relevant to the choice of reflections for refinement. R-factors based on F^2^ are statistically about twice as large as those based on F, and R- factors based on ALL data will be even larger.

### Fractional atomic coordinates and isotropic or equivalent isotropic displacement parameters (Å^2^)

**Table:**

	*x*	*y*	*z*	*U*_iso_*/*U*_eq_	
C1	0.6094 (2)	0.2678 (5)	0.1486 (2)	0.0166 (8)	
H1	0.5909	0.1742	0.1262	0.020*	
C2	0.6825 (2)	0.2881 (6)	0.1666 (2)	0.0202 (8)	
H2A	0.7127	0.2085	0.1556	0.024*	
C3	0.7105 (2)	0.4266 (6)	0.2009 (3)	0.0232 (9)	
H3	0.7593	0.4400	0.2128	0.028*	
C4	0.6652 (2)	0.5450 (5)	0.2173 (3)	0.0209 (8)	
H4	0.6840	0.6372	0.2409	0.025*	
C5	0.5920 (2)	0.5271 (5)	0.1986 (2)	0.0172 (8)	
H5	0.5620	0.6077	0.2090	0.021*	
C6	0.5637 (2)	0.3877 (5)	0.1641 (2)	0.0141 (7)	
C7	0.4861 (2)	0.3647 (5)	0.1464 (2)	0.0136 (7)	
C8	0.4328 (2)	0.4585 (5)	0.1020 (2)	0.0142 (7)	
C9	0.4448 (2)	0.6123 (5)	0.0636 (3)	0.0209 (8)	
H9A	0.4196	0.6942	0.0854	0.025*	
H9B	0.4279	0.6047	0.0087	0.025*	
H9C	0.4946	0.6361	0.0722	0.025*	
C10	0.3698 (2)	0.3785 (5)	0.1048 (2)	0.0144 (7)	
C11	0.2955 (2)	0.4125 (5)	0.0719 (2)	0.0145 (7)	
C12	0.2782 (2)	0.5123 (6)	0.0082 (3)	0.0203 (8)	
H12	0.3143	0.5577	−0.0142	0.024*	
C13	0.2076 (2)	0.5455 (6)	−0.0226 (3)	0.0222 (9)	
H13	0.1969	0.6130	−0.0649	0.027*	
C14	0.1534 (2)	0.4775 (6)	0.0101 (3)	0.0222 (9)	
H14	0.1063	0.4996	−0.0102	0.027*	
C15	0.1695 (2)	0.3761 (6)	0.0734 (3)	0.0234 (9)	
H15	0.1333	0.3289	0.0948	0.028*	
C16	0.2401 (2)	0.3455 (5)	0.1044 (3)	0.0192 (8)	
H16	0.2506	0.2796	0.1474	0.023*	
N1	0.45725 (17)	0.2358 (4)	0.17249 (19)	0.0136 (6)	
N2	0.38684 (17)	0.2464 (4)	0.1472 (2)	0.0148 (6)	
H2B	0.357 (3)	0.167 (6)	0.150 (3)	0.006 (11)*	
Cl1	0.40945 (5)	−0.09017 (12)	0.21857 (7)	0.0219 (3)	
Cu1	0.5000	0.07621 (8)	0.2500	0.0124 (2)	

### Atomic displacement parameters (Å^2^)

**Table:**

	*U*^11^	*U*^22^	*U*^33^	*U*^12^	*U*^13^	*U*^23^
C1	0.0159 (17)	0.0188 (19)	0.0153 (17)	0.0016 (15)	0.0030 (14)	0.0001 (15)
C2	0.0189 (19)	0.026 (2)	0.0158 (18)	0.0058 (16)	0.0036 (15)	0.0032 (16)
C3	0.0146 (17)	0.034 (2)	0.022 (2)	−0.0001 (17)	0.0041 (15)	0.0062 (19)
C4	0.0183 (18)	0.019 (2)	0.026 (2)	−0.0081 (16)	0.0056 (16)	0.0016 (16)
C5	0.0161 (17)	0.0172 (19)	0.0182 (19)	−0.0008 (14)	0.0026 (14)	−0.0008 (15)
C6	0.0112 (15)	0.0173 (18)	0.0142 (17)	0.0011 (13)	0.0029 (13)	0.0014 (14)
C7	0.0120 (16)	0.0168 (18)	0.0124 (17)	0.0000 (14)	0.0031 (13)	−0.0017 (14)
C8	0.0154 (16)	0.0149 (17)	0.0123 (16)	0.0044 (14)	0.0026 (13)	0.0030 (14)
C9	0.0194 (19)	0.0184 (19)	0.025 (2)	0.0036 (15)	0.0048 (16)	0.0115 (16)
C10	0.0120 (16)	0.0172 (18)	0.0131 (17)	0.0047 (14)	−0.0007 (13)	0.0016 (14)
C11	0.0133 (16)	0.0137 (17)	0.0151 (17)	0.0030 (13)	−0.0019 (13)	−0.0022 (14)
C12	0.0175 (18)	0.023 (2)	0.020 (2)	−0.0010 (16)	0.0008 (15)	−0.0013 (16)
C13	0.023 (2)	0.024 (2)	0.0174 (19)	0.0049 (17)	−0.0039 (15)	0.0009 (16)
C14	0.0146 (17)	0.028 (2)	0.021 (2)	0.0052 (16)	−0.0052 (15)	−0.0081 (17)
C15	0.0174 (19)	0.026 (2)	0.027 (2)	0.0030 (16)	0.0034 (16)	0.0011 (18)
C16	0.0170 (18)	0.020 (2)	0.0205 (19)	0.0006 (15)	0.0035 (15)	−0.0003 (16)
N1	0.0135 (14)	0.0122 (15)	0.0147 (15)	0.0021 (12)	0.0013 (12)	0.0027 (12)
N2	0.0120 (14)	0.0134 (15)	0.0185 (16)	−0.0021 (12)	0.0009 (12)	−0.0020 (13)
Cl1	0.0168 (5)	0.0149 (5)	0.0316 (6)	−0.0044 (3)	−0.0034 (4)	0.0033 (4)
Cu1	0.0114 (3)	0.0098 (3)	0.0148 (3)	0.000	−0.0016 (2)	0.000

### Geometric parameters (Å, °)

**Table:**

C1—C2	1.391 (6)	C10—N2	1.355 (5)
C1—C6	1.397 (5)	C10—C11	1.471 (5)
C1—H1	0.9300	C11—C12	1.392 (6)
C2—C3	1.388 (7)	C11—C16	1.399 (6)
C2—H2A	0.9300	C12—C13	1.397 (6)
C3—C4	1.387 (7)	C12—H12	0.9300
C3—H3	0.9300	C13—C14	1.386 (7)
C4—C5	1.393 (6)	C13—H13	0.9300
C4—H4	0.9300	C14—C15	1.393 (7)
C5—C6	1.397 (6)	C14—H14	0.9300
C5—H5	0.9300	C15—C16	1.393 (6)
C6—C7	1.477 (5)	C15—H15	0.9300
C7—N1	1.340 (5)	C16—H16	0.9300
C7—C8	1.418 (5)	N1—N2	1.348 (5)
C8—C10	1.391 (6)	N1—Cu1	1.992 (3)
C8—C9	1.503 (6)	N2—H2B	0.90 (5)
C9—H9A	0.9600	Cl1—Cu1	2.2329 (11)
C9—H9B	0.9600	Cu1—N1^i^	1.992 (3)
C9—H9C	0.9600	Cu1—Cl1^i^	2.2329 (11)
			
C2—C1—C6	120.1 (4)	C8—C10—C11	132.6 (4)
C2—C1—H1	119.9	C12—C11—C16	118.3 (4)
C6—C1—H1	119.9	C12—C11—C10	121.2 (4)
C3—C2—C1	120.3 (4)	C16—C11—C10	120.6 (4)
C3—C2—H2A	119.9	C11—C12—C13	121.1 (4)
C1—C2—H2A	119.9	C11—C12—H12	119.4
C4—C3—C2	119.7 (4)	C13—C12—H12	119.4
C4—C3—H3	120.1	C14—C13—C12	119.8 (4)
C2—C3—H3	120.1	C14—C13—H13	120.1
C3—C4—C5	120.6 (4)	C12—C13—H13	120.1
C3—C4—H4	119.7	C13—C14—C15	119.9 (4)
C5—C4—H4	119.7	C13—C14—H14	120.0
C4—C5—C6	119.8 (4)	C15—C14—H14	120.0
C4—C5—H5	120.1	C14—C15—C16	119.9 (4)
C6—C5—H5	120.1	C14—C15—H15	120.1
C5—C6—C1	119.5 (4)	C16—C15—H15	120.1
C5—C6—C7	120.5 (4)	C15—C16—C11	121.0 (4)
C1—C6—C7	120.0 (4)	C15—C16—H16	119.5
N1—C7—C8	110.3 (3)	C11—C16—H16	119.5
N1—C7—C6	119.4 (4)	C7—N1—N2	106.1 (3)
C8—C7—C6	130.2 (4)	C7—N1—Cu1	129.9 (3)
C10—C8—C7	104.7 (3)	N2—N1—Cu1	122.9 (3)
C10—C8—C9	129.6 (4)	N1—N2—C10	111.8 (3)
C7—C8—C9	125.7 (4)	N1—N2—H2B	123 (3)
C8—C9—H9A	109.5	C10—N2—H2B	124 (3)
C8—C9—H9B	109.5	N1^i^—Cu1—N1	94.1 (2)
H9A—C9—H9B	109.5	N1^i^—Cu1—Cl1	144.42 (10)
C8—C9—H9C	109.5	N1—Cu1—Cl1	92.88 (10)
H9A—C9—H9C	109.5	N1^i^—Cu1—Cl1^i^	92.88 (10)
H9B—C9—H9C	109.5	N1—Cu1—Cl1^i^	144.42 (10)
N2—C10—C8	107.0 (3)	Cl1—Cu1—Cl1^i^	101.34 (6)
N2—C10—C11	120.4 (4)		
			
C6—C1—C2—C3	0.8 (6)	C8—C10—C11—C16	−157.4 (5)
C1—C2—C3—C4	0.0 (7)	C16—C11—C12—C13	0.2 (7)
C2—C3—C4—C5	−0.9 (7)	C10—C11—C12—C13	−179.5 (4)
C3—C4—C5—C6	1.0 (7)	C11—C12—C13—C14	−0.5 (7)
C4—C5—C6—C1	−0.2 (6)	C12—C13—C14—C15	−0.2 (7)
C4—C5—C6—C7	177.9 (4)	C13—C14—C15—C16	1.1 (7)
C2—C1—C6—C5	−0.7 (6)	C14—C15—C16—C11	−1.4 (7)
C2—C1—C6—C7	−178.8 (4)	C12—C11—C16—C15	0.8 (6)
C5—C6—C7—N1	−127.0 (4)	C10—C11—C16—C15	−179.5 (4)
C1—C6—C7—N1	51.1 (5)	C8—C7—N1—N2	−1.2 (4)
C5—C6—C7—C8	54.9 (6)	C6—C7—N1—N2	−179.6 (3)
C1—C6—C7—C8	−127.0 (5)	C8—C7—N1—Cu1	−169.6 (3)
N1—C7—C8—C10	1.3 (4)	C6—C7—N1—Cu1	12.0 (5)
C6—C7—C8—C10	179.5 (4)	C7—N1—N2—C10	0.6 (4)
N1—C7—C8—C9	179.7 (4)	Cu1—N1—N2—C10	170.1 (3)
C6—C7—C8—C9	−2.1 (7)	C8—C10—N2—N1	0.2 (5)
C7—C8—C10—N2	−0.9 (4)	C11—C10—N2—N1	−179.8 (3)
C9—C8—C10—N2	−179.2 (4)	C7—N1—Cu1—N1^i^	47.2 (3)
C7—C8—C10—C11	179.2 (4)	N2—N1—Cu1—N1^i^	−119.6 (3)
C9—C8—C10—C11	0.9 (8)	C7—N1—Cu1—Cl1	−167.7 (3)
N2—C10—C11—C12	−157.6 (4)	N2—N1—Cu1—Cl1	25.5 (3)
C8—C10—C11—C12	22.3 (7)	C7—N1—Cu1—Cl1^i^	−53.6 (4)
N2—C10—C11—C16	22.7 (6)	N2—N1—Cu1—Cl1^i^	139.6 (2)

Symmetry codes: (i) −*x*+1, *y*, −*z*+1/2.
